# Regional Anesthesia-Analgesia in Colorectal Surgical Care for High-Risk Patients With Advanced Myasthenia Gravis: A Case Report

**DOI:** 10.7759/cureus.72842

**Published:** 2024-11-01

**Authors:** Manuj M Shah, Laura C Perez, Leonardo E Garcia, Andres J Gonzalez Salazar, Peter A Najjar

**Affiliations:** 1 Surgery, Johns Hopkins University School of Medicine, Baltimore, USA

**Keywords:** complicated diverticulitis, epidural anesthesia, laparotomy, major open abdominal surgery, myasthenia gravis, neuraxial analgesia, neuraxial anesthesia

## Abstract

Myasthenia gravis (MG) is an antibody-mediated disorder that disrupts postsynaptic acetylcholine receptors with consequent fatigable weakness, bulbar symptoms, and respiratory fragility. MG patients can be challenging to manage during open abdominal surgery given the unpredictable efficacy of neuromuscular and reversal agents and the risk of precipitating an MG crisis. Regional neuraxial anesthesia eliminates the need for these agents and endotracheal intubation. Here, we report the case of a 66-year-old male with a history of advanced MG, vasovagal episodes with bradycardia and asystolic arrest, and complicated diverticulitis who underwent an uncomplicated open sigmoid colectomy achieved with epidural anesthesia-analgesia. Neuraxial anesthesia can be considered and further investigated as an effective approach in optimizing high-risk patients undergoing open laparotomy for colorectal surgical care.

## Introduction

The most common type of neuromuscular transmission disease, myasthenia gravis (MG), is an antibody-mediated autoimmune disorder that disrupts postsynaptic acetylcholine receptors. With the consequent impairment in cholinergic transmission at the neuromuscular junction, MG clinically manifests with fatigable weakness, bulbar symptoms, and respiratory fragility. Recent estimates have shown the incidence of MG to be 4.1 to 30 cases per 1,000,000 with a prevalence of 150 to 200 per 1,000,000 [1]. Given its pathophysiology, MG patients can be challenging to manage from a surgical and anesthetic perspective during open abdominal surgery, especially with the risk of perioperative myasthenic crisis [2]. With unpredictable and varied responses to anesthetic agents, there remains a lack of universal guidelines on how to manage MG patients who are at risk of developing perioperative myasthenic crisis and respiratory failure [2].

Regional neuraxial anesthesia can eliminate the need for these agents and endotracheal intubation. Here, we discuss the colorectal surgical care for a patient with advanced MG and a history of vasovagal syncope with bradycardia and asystolic arrest that increased his risk for perioperative morbidity. Epidural anesthesia-analgesia was employed successfully to optimize his perioperative risk, and an open sigmoid colectomy was carried out without any complications. This case highlights the utility and efficacy of neuraxial anesthesia for colorectal surgical care in such high-risk patients.

## Case presentation

**Figure 1:**
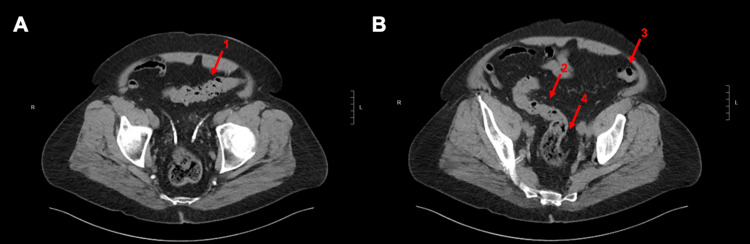
Preoperative computed tomography (CT) scan of the abdomen and pelvis without contrast five months prior to procedure (Panel A, B). Axial views demonstrating colonic diverticulosis involving the sigmoid colon (1, 2, 3) with evidence of contained microperforation. Segmental wall thickening was noted in the sigmoid colon without free fluid or abscess formation (4). The final diagnostic impression was acute diverticulitis affecting the sigmoid colon

A 66-year-old man with a past medical history of MG and primary adrenocortical insufficiency initially presented to an outside hospital with a three-year history of progressive, intermittent lower abdominal pain and diarrhea. Per computed tomography (CT), he was diagnosed with diverticulitis of the sigmoid colon complicated by a contained perforation. Despite completing multiple courses of antibiotics as an outpatient, he continued to experience significant abdominal pain and diarrhea. An interval CT scan showed stable findings without free fluid or abscess formation (Figure 1). The patient was seen in the clinic and scheduled for sigmoid colectomy and colostomy creation in close coordination with anesthesia for perioperative planning.

His past medical history was also notable for iron deficiency anemia, anxiety, asthma, mild aortic stenosis, nephrolithiasis, hypertension, previous saddle pulmonary embolism (three years prior), psoriasis, pulmonary hypertension, sleep apnea, temporomandibular joint pain, type 2 diabetes mellitus, and allergies to ciprofloxacin, gadobutrol, and propranolol. He utilized a bilevel positive airway pressure (BiPAP) machine at home for 18 hours daily as noninvasive ventilatory support; his daily medication regimen included prednisone 10 mg daily, pyridostigmine 120 mg three times daily in addition to 360 mg nightly, rituximab infusions every eight weeks, lisinopril 20 mg daily, apixaban 2.5 mg twice daily, and omeprazole 20 mg daily. The patient’s surgical history included a basal cell carcinoma excisional biopsy and nephrolithiasis surgery. He had no history of abdominal surgery. His pulmonary function tests performed one month prior to surgery showed a forced expiratory volume to forced vital capacity ratio (FEV1/FVC) of 0.81 (2.92 L/3.61 L) with normal flow volume loop patterns and no obstructive ventilatory defect upon forced expiration. The preoperative electrocardiogram was overall reassuring, with moderate evidence of right ventricular hypertrophy and inferior ST changes. Given his extensive history, a decision was made to preadmit the patient to the intensive care unit a day prior to his scheduled surgery.

Upon admission, vital signs were stable with an oxygen saturation of 98-100% on BiPAP, a respiration rate of 22 breaths per minute, and preoperative hemoglobin of 11.7 mg/dL. The patient continued receiving prednisone and pyridostigmine; all other home medications were paused. Upon an attempt to establish peripheral intravenous (IV) access overnight, the patient experienced a profound vagal episode with bradycardia that progressed to asystole with loss of pulses upon palpation; he lost consciousness and had agonal breathing. Cardiopulmonary resuscitation was initiated with the performance of chest compression for 20-30 seconds and the placement of defibrillation pads. Within a minute, his heart rate rapidly climbed back to 40-50 beats per minute; his pulse soon became detectable and palpable, and his ventilation improved dramatically. Over the next five to 10 minutes, his mental status improved back to baseline, and the patient was able to fluently converse with orientation to self.

Given the patient’s six-year history of MG, need for ventilatory support, and recent asystolic arrest, we were concerned that general anesthesia would pose a greater risk of respiratory dysfunction and an extended hospital course in the intensive care unit postoperatively; consequently, we elected and planned for neuraxial anesthesia with spontaneous noninvasive ventilatory support via his home BiPAP machine and supplemental oxygen by placing an oxygen mask near the machine’s air inlet. Approximately 10 hours after the vasovagal episode with asystolic arrest, the patient was brought to the operating room for an open sigmoid colectomy with end colostomy creation. With the patient sitting upright, 3 mL of 1% lidocaine was injected in the right paramedian region, and a 17-gauge Tuohy needle was advanced to the epidural space at the T11-12 level, with confirmation by saline loss-of-resistance, to a depth of 5.8 cm, allowing for placement of a 19-gauge wire-reinforced epidural catheter. Ten milliliters of a 2.4% lidocaine, morphine 0.4 mg/mL, and epinephrine 5 mcg/mL solution were administered through the epidural catheter in divided doses. The patient received IV glycopyrrolate (0.4 mg) for bradycardia prophylaxis as well as dexmedetomidine (85 mcg, 0.9 mcg/kg) for mild sedation before incision. Injection of hydrocortisone sodium succinate 100 mg was given in addition to prophylactic antibiotics, namely cefazolin 2 g and metronidazole 500 mg. Continuous epidural anesthesia was maintained with 0.25% bupivacaine (10 mL/hr).

While the patient was awake and positioned in lithotomy, a lower midline incision was used to enter the abdomen with subsequent mobilization of the left colon and colon mesentery and lysis of adhesions. The inferior mesenteric artery was divided with a Vicryl tie, the descending colon was divided with a gastrointestinal anastomosis stapler, the upper rectum was divided with a curved cutter stapler and oversewn, and an end colostomy was created in the left lower quadrant without complication. During the procedure, the patient received an additional epidural bolus of 0.5% bupivacaine (3 mL) for discomfort, an IV bolus of glycopyrrolate (0.2 mg) for bradycardia, and IV boluses of dexmedetomidine (66 mcg) and ketamine (50 mg) for moderate sedation. In addition, he received norepinephrine (0.28 mg) in dextrose 5% water at 0.03 mcg/kg/hr to support his low blood pressure. Total operating room time was 140 minutes, and the patient received a total of 1L of PlasmaLyte A infusion IV with 250 mL of urine output and a 50 mL estimated blood loss. At the end of the case, a 19 French Jackson-Pratt surgical drain was placed in the pelvis along with an IVAC^TM^ PCAM^TM^ syringe pump over the midline incision. Immediately postoperatively, the patient’s vital signs were stable with an oxygen saturation of 99%, respiration rate of 22 breaths per minute, heart rate of 49 beats per minute, and blood pressure of 92/47 mmHg, and he was transferred to the intensive care unit for recovery.

Postoperatively, the patient experienced two additional vasovagal episodes with asystole arrest, both with near-immediate return of spontaneous circulation without any need for cardiopulmonary resuscitation or medication. IV heparin was restarted. The patient experienced a mild postoperative ileus that resolved in the days following routine supportive care. With subsequent tolerance of a regular diet, ability to ambulate, and resolution of postoperative symptoms, his remaining hospital course was unremarkable. The patient continued to be hemodynamically stable and comfortable, and he was discharged home eight days after the surgery.

## Discussion

It remains challenging to optimize patients with advanced MG during major open colorectal surgery. Preoperative intubation and anesthesia procedures often require neuromuscular blocking medications. However, these agents are generally avoided in MG patients, as depolarizing agents such as succinylcholine are essentially ineffective, and nondepolarizing agents have unpredictable efficacy and may exacerbate MG symptoms [3,4]. If deemed appropriate and utilized, these agents can be theoretically reversed with sugammadex, but this strategy has shown limited reliability in MG patients [5].

To help mitigate the concerns with advanced MG in a perioperative setting, regional anesthesia can be utilized to eliminate the need for neuromuscular blocking agents when pursuing open abdominal surgery [6]. A 1994 comparative study investigating the feasibility and efficacy of awake epidural anesthesia in open colectomies revealed that despite having more comorbidities, high-risk patients could safely undergo awake epidural anesthesia with comparable survival and rates of complications [7,8]. Thus, given our patient’s history, respiratory fragility, and prior episodes of vasovagal episodes with asystolic arrest, we opted to employ epidural anesthesia to try to minimize intraoperative and postoperative complications. Epidural anesthesia offers the added advantage of postoperative analgesia, which is key in optimizing respiratory function, and decreases the need for high systemic doses of postoperative opioids, as demonstrated by previous studies of successful transsternal thymectomies and cesarean sections under neuraxial anesthesia in MG patients [9-11].

Other important considerations for MG patients undergoing major abdominal surgery include the risk of postoperative respiratory failure, maintenance of outpatient medications, and effects of chronic glucocorticoid use. With respiratory failure being a key characteristic of myasthenic crises, we recognize that MG patients have an increased risk of prolonged postoperative mechanical ventilation [12]. Consistently decreased FVC and severe bulbar symptoms should signal providers to closely monitor patient respiratory function and quickly initiate ventilatory support and intubation when needed [13]. Furthermore, when using neuraxial anesthesia, providers should be careful in administering too high of a block such that intercostal muscle movement and respiratory function may be compromised [14]. With our patient, we placed the epidural catheter at the T11-12 level and maintained respiratory status by continuing his noninvasive ventilatory support intra- and postoperatively with close monitoring. Additionally, the sympathectomy brought on by epidural anesthesia may warrant the need for blood pressure and cardiovascular support, as evidenced by the use of vasopressors intraoperatively for our case. One of the mainstays of treatment for MG patients is an anticholinesterase agent, such as pyridostigmine or neostigmine; because MG patients can be unpredictably sensitive to the sudden discontinuation of these medications, they are most often continued up to the time of the patient’s operation, as was done with our patient [2]. Lastly, autoimmune conditions often coexist, and MG patients can have synchronous adrenocortical insufficiency. Moreover, glucocorticoids are often used in the treatment of MG, so patients are at increased risk for developing secondary adrenocortical insufficiency and may require the administration of stress-dose steroids. Current guidelines suggest that individuals chronically taking at least 5 mg of prednisone daily should receive a stress dose before surgery, as shown by this patient having his 10 mg daily dose increased to a one-time 20 mg dose the day before his operation [2].

Neuraxial anesthesia has been previously demonstrated with success in comorbid patients undergoing other major thoracic and abdominal surgeries, such as inguinal hernia repair, thymectomy, cholecystectomy, mammaplasty, and partial colectomies [15-22]. Here, we report a successful open sigmoid colectomy under epidural anesthesia for complicated diverticulitis in a high-risk patient with severe MG, respiratory fragility, and a history of cardiac dysrhythmias. Despite our patient’s high-risk features for an open abdominal procedure, the utilization of epidural anesthesia allowed us to perform the surgery successfully with a sufficient sensory and motor block while avoiding the systemic effects of general anesthesia, tracheal intubation, respiratory compromise, and the need for systemic postoperative opioids for pain control.

## Conclusions

With this patient’s history, respiratory fragility, and prior vasovagal episodes with asystolic arrest, this case highlights the efficacy of neuraxial anesthesia during major open abdominal surgery to minimize perioperative complications, namely MG crises. Given MG patients’ increased risk of prolonged mechanical ventilation postoperatively, it is important to carefully select the level of anesthetic block to avoid respiratory compromise. Additionally, MG patients are unpredictably sensitive to discontinuation of their medications, so these should be continued through the perioperative period. In advanced MG patients, surgical and anesthetic management should be carefully discussed and include evaluating the need for sedation, the use of neuraxial anesthesia, and the optimization of pertinent medications to support a safe, streamlined operation and recovery.
